# Radiation Sensitization of Basal Cell and Head and Neck Squamous Cell Carcinoma by the Hedgehog Pathway Inhibitor Vismodegib

**DOI:** 10.3390/ijms19092485

**Published:** 2018-08-23

**Authors:** Stephanie Hehlgans, Patrick Booms, Ömer Güllülü, Robert Sader, Claus Rödel, Panagiotis Balermpas, Franz Rödel, Shahram Ghanaati

**Affiliations:** 1 Department of Radiotherapy and Oncology, Medical Center Goethe-University Frankfurt am Main, 60590 Frankfurt am Main, Germany; Oemer.Guelluelue@kgu.de (Ö.G.); Claus.Roedel@kgu.de (C.R.); Panagiotis.Balermpas@kgu.de (P.B.); Franz.Roedel@kgu.de (F.R.); 2 Department for Oral, Cranio-Maxillofacial and Facial Plastic Surgery, Frankfurt Orofacial Regenerative Medicine (FORM), Medical Center Goethe-University Frankfurt am Main, 60590 Frankfurt am Main, Germany; Patrick.Booms@kgu.de (P.B.); Robert.Sader@kgu.de (R.S.); Shahram.Ghanaati@kgu.de (S.G.)

**Keywords:** basal cell carcinoma, head and neck squamous cell carcinoma, hedgehog signaling pathway, radiotherapy resistance, vismodegib (GDC-0449)

## Abstract

Vismodegib, an inhibitor of the Hedgehog signaling pathway, is an approved drug for monotherapy in locally advanced or metastatic basal cell carcinoma (BCC). Data on combined modality treatment by vismodegib and radiation therapy, however, are rare. In the present study, we examined the radiation sensitizing effects of vismodegib by analyzing viability, cell cycle distribution, cell death, DNA damage repair and clonogenic survival in three-dimensional cultures of a BCC and a head and neck squamous cell carcinoma (HNSCC) cell line. We found that vismodegib decreases expression of the Hedgehog target genes glioma-associated oncogene homologue (GLI1) and the inhibitor of apoptosis protein (IAP) Survivin in a cell line- and irradiation-dependent manner, most pronounced in squamous cell carcinoma (SCC) cells. Furthermore, vismodegib significantly reduced proliferation in both cell lines, while additional irradiation only slightly further impacted on viability. Analyses of cell cycle distribution and cell death induction indicated a G1 arrest in BCC and a G2 arrest in HNSCC cells and an increased fraction of cells in SubG1 phase following combined treatment. Moreover, a significant rise in the number of phosphorylated histone-2AX/p53-binding protein 1 (γH2AX/53BP1) foci in vismodegib- and radiation-treated cells was associated with a significant radiosensitization of both cell lines. In summary, these findings indicate that inhibition of the Hedgehog signaling pathway may increase cellular radiation response in BCC and HNSCC cells.

## 1. Introduction

Non-melanoma skin cancer, or keratinocyte carcinoma, is the most common malignancy among Caucasians and its incidence continues to rise annually [1]. This entity comprises basal cell carcinoma (BCC) and squamous cell carcinoma (SCC), together with a host of rare tumors [2,3]. BCC arises from abnormal growth of the skin’s basal cells, which line the deepest layer of the epidermis, with ultraviolet light exposure to constitute an essential risk factor in the development of malignancy [4]. The mechanisms of origin of BCCs have been extensively analyzed within the last decades. Briefly, a reactivation of the hedgehog (Hh) signaling pathway has been involved in tumorigenesis in human BCC [5]. In an unaffected individual, the Hh signaling pathway is inhibited by patched homologue 1 (PTCH1) and gets activated upon binding of Hh ligand to its transmembrane receptor smoothened (SMO). SMO is a G-protein-coupled receptor (GPCR)-like receptor that transfers signals through various proteins resulting in an increased expression of target genes, such as glioma-associated oncogene homologue zinc-finger transcription activator (GLI1) [6]. An estimated 80–90% of sporadic BCC tumors have PTCH1 mutations, whereas 10% harbor SMO mutations. In either scenario, these alterations lead to unopposed SMO signaling and BCC development [7,8].

Recent data indicate that the inhibitor of apoptosis protein (IAP) Survivin comprises a novel target of the Hh/GLI signaling pathway [9]. Direct proof of GLI2 transcription factor binding to the Survivin promoter, promoter activity assays and Survivin downregulation upon Hh/GLI signaling inhibition by SMO inhibitor cyclopamine, or GLI inhibitor GANT61 identified Survivin as a transcriptional target of GLI2 in a variety of tumor cells of different origin as well as in a murine tumor model [9]. A hampered Survivin expression following SMO inhibition has further been described for malignant pleural mesothelioma, an effect rescued by overexpression of GLI1, suggesting an involvement of GLI1 transcription factor [10]. Importantly, Survivin is frequently overexpressed in malignant cells, confers chemo- and radiation resistance by multiple pathways including inhibition of Caspase-3 and -7 activity and p53-mediated apoptosis [11] and has been associated with poor clinical outcome [12].

In 2012, the Hh pathway inhibitor vismodegib (GDC-0449), an oral small molecule inhibitor of SMO was the first drug to gain U.S. Food and Drug Administration approval for treatment of advanced BCC, metastasized BCC, relapsed BCC after surgery, or BCC that cannot be treated with surgery or radiation [13,14]. Vismodegib monotherapy has been shown to contribute to significant response rates between 30% and 43% (phase 1 study) among patients with advanced BCC [15]. Moreover, preclinical and clinical data indicate that an aberrant Hh signaling [16,17], mutations in PTCH1 and SMO and overexpression of components of the Hh pathway all correlate with poor clinical outcome in gastric [18], esophageal cancer [19] and in head and neck SCC (HNSCC) [20,21]. In line with that, inhibition of Hh pathway if combined with radiation or radiochemotherapy (RCT), has been shown to result in improved therapeutic responses and survival, without increased unexpected side effects in a variety of preclinical models and clinical reports including non-small cell lung cancer [22], esophageal cancer [19], and HNSCC [16]. Moreover, combining vismodegib and radiotherapy (RT) displays a beneficial therapeutic effect on BCC [23,24] as shown by our group for locally advanced tumors of the head and neck region [25]. In the present study, we aimed to confirm a radiation sensitizing effect of vismodegib in a BCC and an HNSCC cell line.

## 2. Results

### 2.1. Impact of Vismodegib Concentration and Treatment Time on Cell Proliferation/Viability

**Figure 1:**
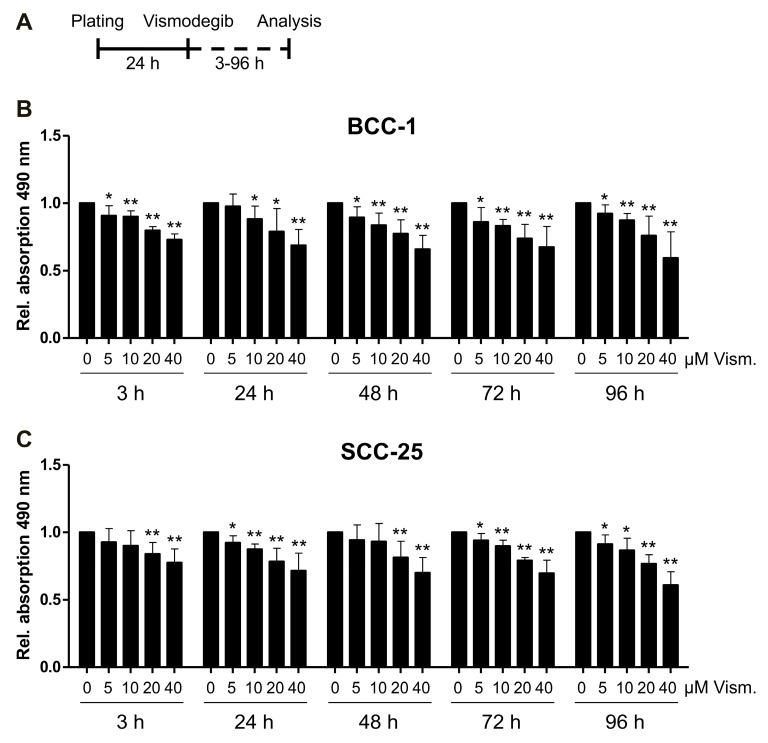
Vismodegib reduces the number of viable BCC-1 and SCC-25 cells. Relative quantification of cellular metabolic activity (i.e., number of viable cells) was performed for cells treated with vismodegib compared to dimethyl sulfoxide (DMSO) controls using a CellTiter 96^®^ Aqueous One Solution Cell Proliferation (MTS) Assay. (**A**) Time schedule of vismodegib treatment and measurement of colorimetric cell proliferation assay. BCC-1 or SCC-25 cells were plated 24 h before treatment with vismodegib for 3 to 96 h before analysis. Metabolic activity is expressed as relative absorption at 490 nm. This assay was carried out in five independent experiments (each in quadruplicate) for BCC-1 (**B**) and SCC-25 (**C**) cells. The metabolic activity of controls was set to 1.0 (i.e., reference value) as indicated. Statistical significance was assessed by *t*-test; * *p <* 0.05, ** *p <* 0.01 (vismodegib- versus DMSO-treated cells). BCC, basal cell carcinoma; Rel., relative; SCC, squamous cell carcinoma; Vism., vismodegib.

In order to identify suitable vismodegib concentrations and treatment schedules, we treated BCC-1 and SCC-25 cells with increasing amounts of vismodegib ranging from 5 to 40 µM for 3 to 96 h and applied a colorimetric cell proliferation assay (MTS) (Figure 1A). Cellular proliferation/viability was significantly decreased in a concentration dependent manner within 3 h of treatment with 5 to 40 µM vismodegib, while elongated treatment time up to 96 h only slightly further decreased the cell proliferation/viability in BCC-1 and SCC-25 cells (Figure 1B,C). Notably, we did not reach IC_50_ values indicating the necessity to further increase the therapeutic effects by combining with additional treatment options.

### 2.2. Vismodegib Decreases Hh Signaling Target Gene GLI1 and Survivin Expression in a Cell Line-Dependent Manner

**Figure 2:**
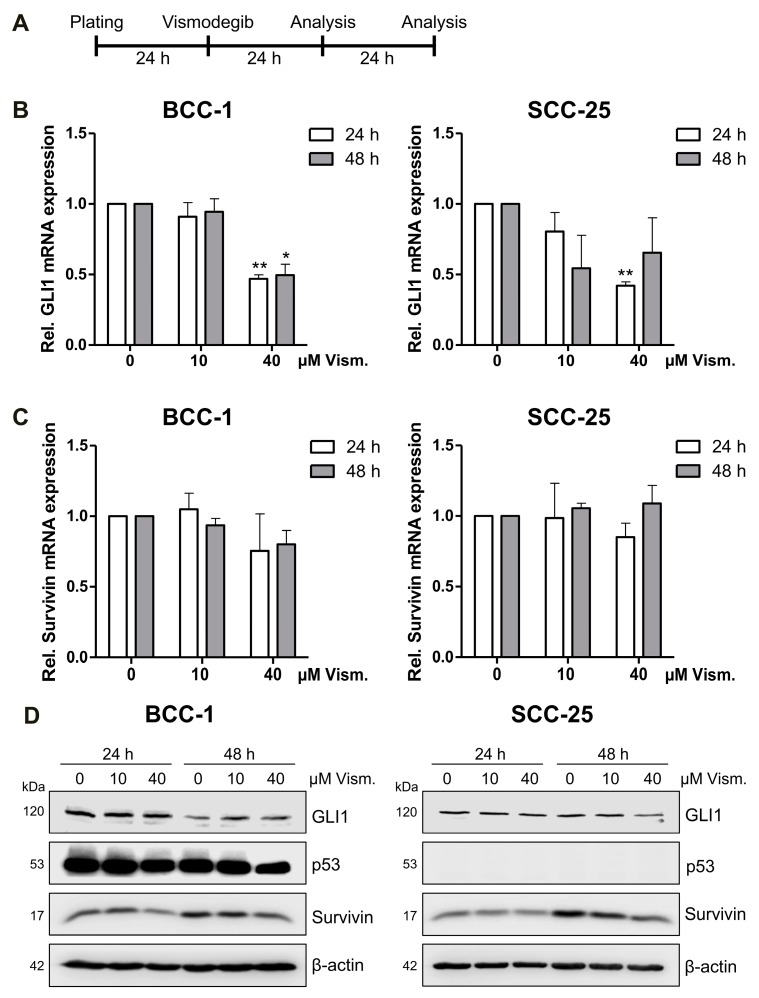
Vismodegib decreases hedgehog (Hh) target gene glioma-associated oncogene homologue 1 (GLI1) and Survivin expression. (**A**) Time schedule of vismodegib application and RNA/protein extraction for analysis. BCC-1 or SCC-25 cells were plated 24 h before treatment with 10 or 40 µM vismodegib or with DMSO as control for 24 h or 48 h before analysis. (**B**) mRNA expression for GLI1 and Survivin (**C**) relative to DMSO-treated controls. *n* = 2 (in duplicate); * *p <* 0.05, ** *p <* 0.01 (vismodegib- versus DMSO-treated cells, *t*-test). (**D**) Representative Western blots from at least two independent experiments, including detection of p53 expression in the cell lines. β-actin served as loading control. Rel., relative; Vism., vismodegib.

To confirm a vismodegib-mediated inhibition of Hh signaling, we applied quantitative real-time PCR and immunoblotting monitoring the expression of Hh target genes GLI1 and Survivin at 24 h and 48 h after vismodegib treatment (Figure 2 and Figure S1). GLI1 mRNA expression was significantly decreased after 24 h of treatment with 40 µM vismodegib in both cell lines while BCC-1 cells further revealed slightly but significantly reduced GLI1 mRNA levels after 48 h (Figure 2B). The low effects of Hh inhibition in both BCC-1 and SCC-25 cells may be attributed to a weak expression of GLI1 protein. Therefore, we compared levels of detection to a HT-29 colorectal cell line, reported to express higher amounts of the protein. As depicted in Figure S2, we detected a pronounced GLI1 band in the HT-29 samples, but a lesser staining in BCC-1 and SCC-25 cells in favor of a weak responsiveness to Hh inhibitor in the latter cell lines. Concerning the expression of Survivin (BIRC5), we observed a slight reduction after 24 and 48 h of vismodegib treatment in the BCC-1 cell line, while survivin expression was not affected in SCC-25 cells on the level of RNA expression (Figure 2C). According to Western blotting (Figure 2D) and densitometric analysis (Figure S1A), vismodegib treatment decreased both GLI1 protein levels in BCC-1 and SCC-25 cells. Notably, Survivin protein expression was slightly but significantly reduced on the protein level (Figure S1B) in SCC-25 cells indicating a putative non-transcriptional regulation following vismodegib treatment.

### 2.3. Vismodegib Treatment and Irradiation Modulate Cell Viability, Cell Cycle Distribution and Cell Death

**Figure 3:**
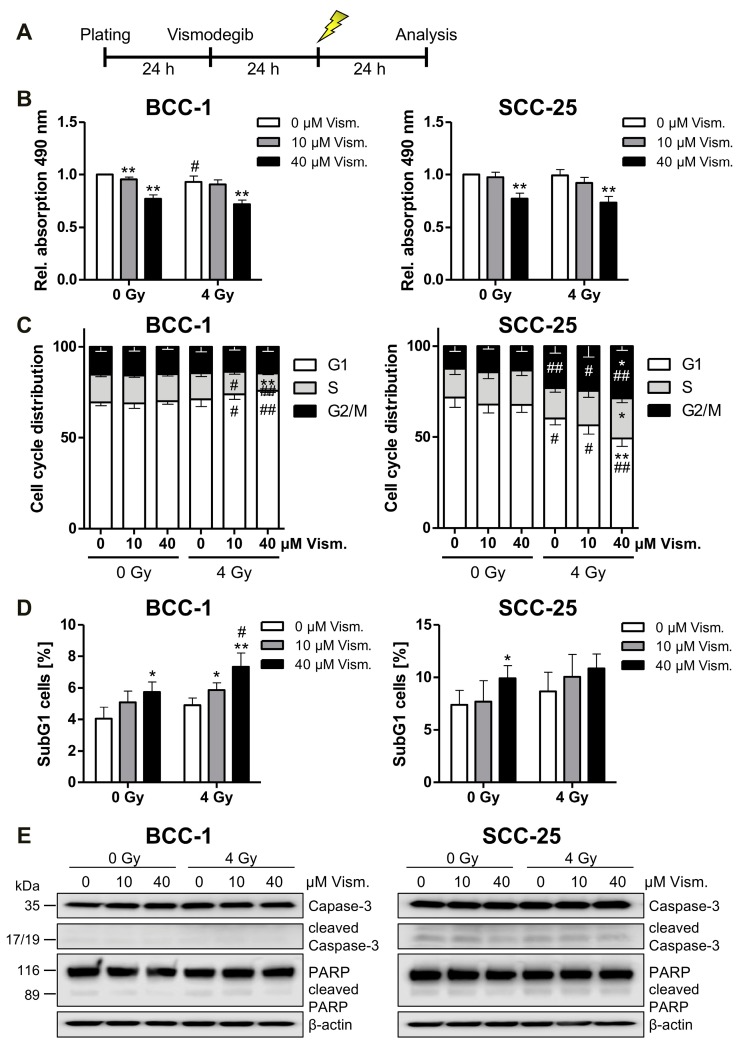
Vismodegib and irradiation modulate cell viability, cell cycle distribution and SubG1 cell fraction content. BCC-1 and SCC-25 cells were pretreated for 24 h with indicated concentrations of vismodegib or DMSO as control before a 4 Gy irradiation (**A**). At 24 h after irradiation, proliferation/viability was measured with a CellTiter 96^®^ Aqueous One Solution Cell Proliferation (MTS) Assay (**B**). Cell cycle distribution (**C**) and SubG1 cell fraction (**D**) were analyzed after propidium iodide staining by flow cytometric quantification. Caspase 3 and PARP expression/cleavage was detected by Western blotting (*n* = 2) with β-actin as loading control (**E**). Data given in (**B**–**D**) are shown as means + SD from four independent experiments with quadruplicates (MTS assay, (**A**)) or duplicates (flow cytometry (**B**,**C**)). Differences were considered as statistically significant when * *p <* 0.05 or highly significant when ** *p <* 0.01; vismodegib- versus DMSO-treated cells (*t*-test). Significant differences between irradiated and non-irradiated cells are indicated as follows: ^#^ *p <* 0.05, ^##^ *p <* 0.01 (*t*-test). Gy, Gray; PARP, poly ((adenosine diphosphate)ADP-ribose) polymerase; Rel., relative; Vism., vismodegib.

To analyze vismodegib- and irradiation-dependent viability/proliferation, MTS assays were performed according to the schedules depicted in Figure 3A. Following combined vismodegib/radiation treatment, we only observed a slight impact of irradiation with 4 Gy on both cell lines investigated (Figure 3B). Since a cell cycle arrest may underlie these findings, we next assayed cell cycle distribution of vismodegib-treated and irradiated BCC-1 cells and found a decreased number of S phase cells, corresponding to an increased fraction of cells in the G1 phase after combined treatment (Figure 3C and Figure S3). In contrast, analysis of SCC-25 cells revealed a G2 phase arrest upon combined-modality treatment (Figure 3C) that might be associated with the lack of p53 expression in the cell line as depicted in Figure 2D. Additionally, we checked the SubG1 cell population to determine cell death induction upon Hh inhibition and irradiation. Vismodegib treatment significantly enhanced the fraction of cells in the SubG1 phase in both cell lines, although at a low level. Additional irradiation with 4 Gy further increased SubG1 cell counts with significant values in BCC-1 cells treated with 40 µM vismodegib (Figure 3D and Figure S3). Finally, Western blot analyses revealed neither increased Caspase-3 nor Poly ((adenosine diphosphate)ADP-ribose) polymerase (PARP) cleavage upon vismodegib and irradiation treatment at the investigated time points (Figure 3E).

### 2.4. Vismodegib Increases Radiation-Induced DNA Damage of BCC-1 and SCC-25 Cells

**Figure 4:**
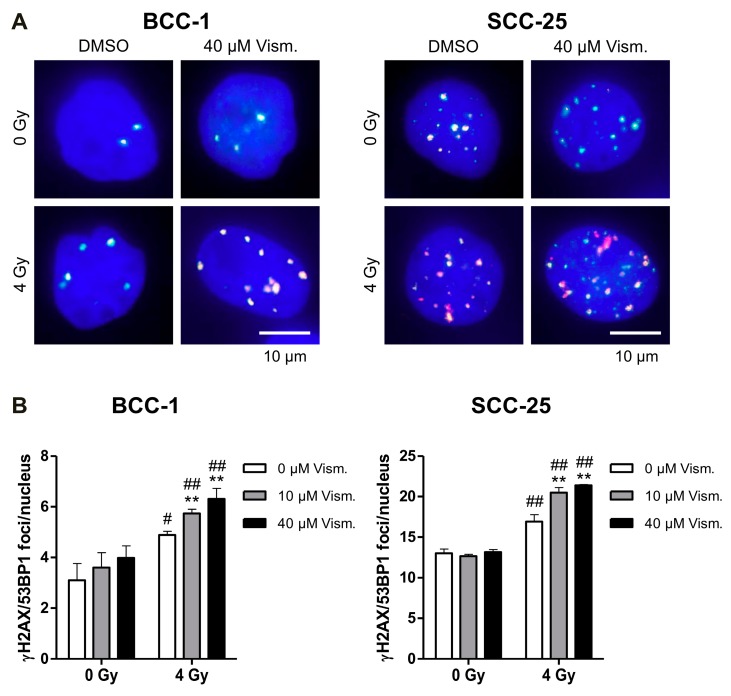
Vismodegib increases radiation-induced γH2AX/53BP1 nuclear foci. BCC-1 or SCC-25 cells were treated for 24 h with 10 or 40 µM vismodegib or with DMSO as control followed by irradiation with 4 Gy. (**A**) Representative images of phosphorylated gamma histone-2AX (γH2AX)/p53-binding protein 1 (53BP1) foci from non-irradiated and 4 Gy-irradiated DMSO control versus 40 µM vismodegib-treated cells are shown. Nuclei were counterstained with DAPI. Scale bar, 10 µm. (**B**) Quantification of persistent γH2AX/53BP1 foci in vismodegib treated and irradiated BCC-1 (left panel) and SCC-25 (right panel) cells. Bars represent means + SD from three independent experiments performed in duplicate. ** *p <* 0.01 vismodegib- versus DMSO-treated cells and ^#^ *p <* 0.05, ^##^ *p <* 0.01 4 Gy versus non-irradiated cells (*t*-test). Gy, Gray; Vism., vismodegib.

Since the Hh/GLI signaling pathway and the target gene Survivin have been described to be involved in DNA repair mechanisms [26,27,28], we investigated the impact of vismodegib on DNA double-strand break (DSB) repair by quantification of γH2AX/p53-binding protein 1 (53BP1) foci detection (Figure 4A). While vismodegib monotherapy does not increase γH2AX/53BP1 nuclear foci in non-irradiated BCC-1 or SCC-25 cells, a combination of vismodegib and 4 Gy-irradiation significantly enhanced the number of persisting (24 h) foci in a concentration-dependent manner (Figure 4). Notably, basal γH2AX/53BP1 foci levels are much higher in SCC-25 HNSCC as compared to BCC-1 cells (Figure 4).

### 2.5. Vismodegib Radiosensitizes BCC-1 and SCC-25 Cells

**Figure 5:**
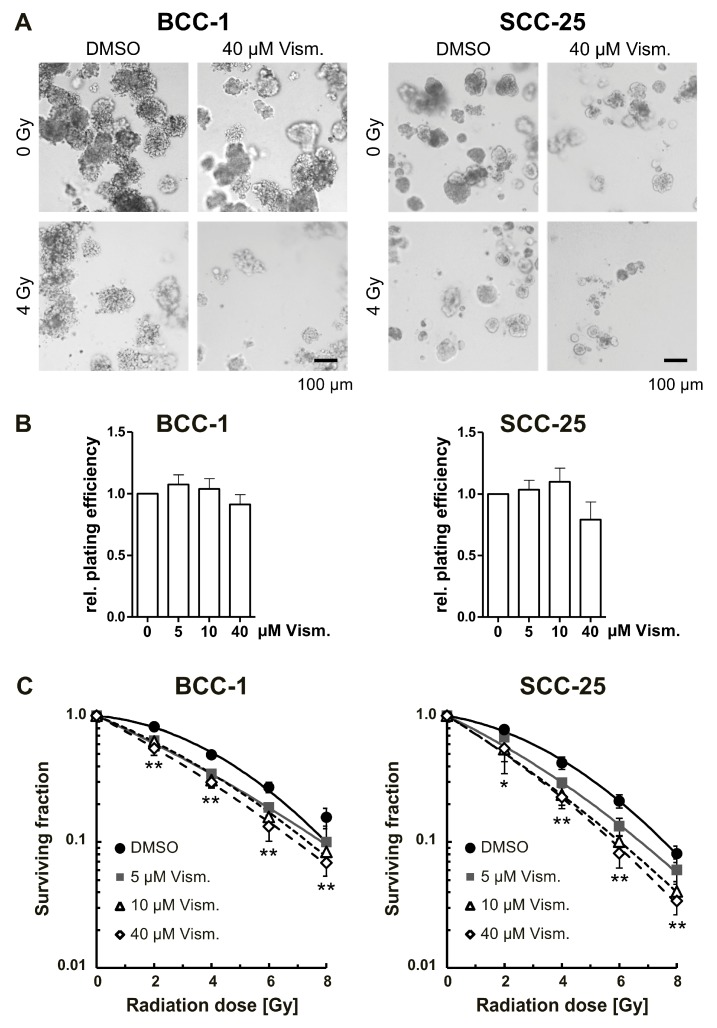
Vismodegib sensitizes basal and squamous cell carcinoma cells to ionizing radiation in a 3D clonogenic assay. BCC-1 and SCC-25 cells were plated as single cells in a three-dimensional (3D) laminin-rich extracellular matrix and were treated with vismodegib 24 h before irradiation with 0 to 8 Gy, single doses. (**A**) 7 days after plating, representative images of 3D grown colonies were acquired. Scale bar, 100 µm. (**B**) Colonies were counted microscopically from three independent experiments performed in triplicate. Basal plating efficiencies of cells treated with 5 to 40 µM vismodegib, are shown relative to DMSO-treated control cells. (**C**) Surviving fractions and radiation survival curves were calculated as described in the Materials and Methods section. Values represent means ± SD (*n* = 3). * *p <* 0.05, ** *p <* 0.01; vismodegib-treated cells versus DMSO control (*t*-test). Gy, Gray; Vism., vismodegib.

Finally, we evaluated long-term effects of vismodegib treatment in combination with irradiation in a more physiological cell culture model by applying 3D clonogenic survival assays (Figure 5A) [29,30]. Single vismodegib treatment does not impact on the basal plating efficiency of BCC-1 and SCC-25 cells (Figure 5B). In contrast, the drug significantly radiosensitized BCC-1 and SCC-25 cells in a concentration-dependent manner (Figure 5C).

Radiation response variables and significances are summarized in Table 1 and Table S1. The radiation-induced cytotoxicity enhancement factors at 50% cell survival comparing 5, 10 or 40 µM vismodegib versus dimethyl sulfoxide (DMSO) control were 1.51, 1.45, and 1.73 for BCC-1 cells, and 1.48, 1.79, and 1.81 for SCC-25 cells (Table 1)—indicating clear radiation sensitizing effects.

## 3. Discussion

BCC and HNSCC tumorigenesis have been shown to be associated with an aberrant canonical Hh/GLI signaling pathway, mostly due to loss-of-function mutations in PTCH1, or gain-of-function mutations in SMO [6,21,31]. Vismodegib, an approved drug for locally advanced or metastatic BCC, antagonizes SMO receptor activation and reduces GLI expression by means of suppressor of fused (SUFU) inhibitor [32]. First preclinical and clinical data have reported the feasibility of using vismodegib in combination with radiation therapy in BCC patients [23,24,25]. Hence, we aimed to investigate, whether inhibition of the Hh pathway using vismodegib might be feasible to overcome BCC or HNSCC therapy resistance and to unravel underlying mechanisms.

Our findings indicate that BCC-1 cells and SCC-25 cells treated with vismodegib are less resistant to irradiation and less viable when compared to untreated controls concomitant with a slight GLI1 and Survivin downregulation, cell cycle modulation and perturbed DNA repair. In more detail, vismodegib-mediated Hh inhibition significantly decreased the number of viable BCC-1- and SCC-25 cells, as shown by a metabolic MTS assays (Figure 1). These observations are in line with data from Steg and colleagues [33] and Wu et al. [34], reporting similar results after SMO inhibition with cyclopamine in human pancreatic cancer cells, or with vismodegib in colon cancer cells, respectively. In the present study, addition of irradiation only slightly decreased the cellular viability (Figure 3), indicating a non-synergistic effect of combined modality treatment at early times. Again, these findings are in line with a recent report by Gonnissen et al. showing no impact of radiation therapy on proliferation of prostate cancer cells pretreated with 1 or 10 µM vismodegib [35]. Thus, one may assume that a therapeutic effect of vismodegib treatment may not be associated with a short-term inhibition of viability/proliferation but may cover other mechanisms of toxicity. Against this background, we investigated the effect of combined vismodegib and radiation treatment on long-term clonogenic radiation survival performed in more physiologic 3D cell culture approaches (Figure 5). The development of 3D cell culture models, including laminin-rich extracellular matrix (lrECM) as for the first time applied in the present study, has shown that these assays provide results with a higher similarity to in vivo data than those obtained from conventional 2D cell cultures [36,37,38]. Responses of 3D grown cells to drug treatment or ionizing radiation have especially revealed an increased therapy resistance that reflects the in vivo situation to a higher extent [36,39]. Putative underlying mechanisms include morphological changes, altered cell-cell and cell-extracellular matrix contacts, activating cell adhesion molecules and different intracellular signaling pathways, differential gene expression or epigenetic changes [36,38,40]. In these more physiological conditions, our data revealed a concentration-dependent radiosensitization of both BCC-1 and SCC-25 cells. Sensitizer enhancement-ratios of approximately 1.5 for the relatively low concentration of 5 µM vismodegib (Table 1) suggest that combination therapy impacts on clinically relevant clonogenic endpoints. Similar effects using conventional 2D assays on cyclopamine-treated human HN11 and TU167 HNSCC [16] and Colo-357 and SW-1990 pancreatic adenocarcinoma cells further support this assumption [41].

On a molecular level, vismodegib treatment slightly decreased GLI1 mRNA and protein expression in both cell lines, while we observed Survivin downregulation upon vismodegib treatment only on the protein level most pronounced at a concentration of 40 µM (Figure 2 and Figure S1). Indeed, a multitude of post-translational modifications, including phosphorylation, acetylation and ubiquitination, have been reported to mediate Survivin′s stability and functions (reviewed in Reference [42]) that may account for the role of this protein in vismodegib-mediated cytotoxicity.

Moreover, cell cycle analysis revealed a G1 arrest in BCC-1 cells and a G2 arrest in SCC-25 cells upon combined vismodegib and radiation treatment, but not after sole vismodegib application (Figure 3). GLI translocation to the nucleus has been reported to promote cell cycle progression [43], and inhibition of GLI by GANT61 was shown to induce a G1 arrest in 22Rv1—but not in PC3 and DU145 pancreatic cancer cells, concomitant with radiosensitization only in p53 wildtype 22Rv1 cells [35]. SCC-25 cells used in our experiments are heterozygous for a p53 mutation [44,45], while the p53 status of BCC-1 is not known at present. Against this background, we performed WB analysis indicating that SCC-25 do not display detectable levels of the p53 protein, while BCC-1 revealed a pronounced detection of the protein (Figure 2D). This may indicate that p53 expression in BCC-1 may be sufficient to modulate e.g., induction of a G1 arrest [46] upon vismodegib/irradiation treatment.

Our results showed that cell death was slightly (but significantly) induced upon Hh inhibition, and irradiation as indicated by an elevated SubG1 fraction 24 h after irradiation and 48 h after vismodegib application (Figure 3D). Cleavage of PARP or Caspase 3 as typical indicators for a canonical apoptotic cell death induction was not evident in our models. This may indicate that alternative cell death mechanisms could contribute to the observed radiosensitizing effect. Although not in the focus of the present experimental setup, further analyses will focus on e.g., non-caspase dependent necroptosis [47], after combined vismodegib/radiation treatment. These findings are in line with data from Shi and colleagues, indicating a lack of PARP and Caspase 3 cleavage in malignant pleural mesothelioma cells upon treatment with the SMO inhibitor HhAntag [10]. Notably, the group further reported on a decreased mRNA and protein expression of Survivin and decreased clonogenic survival similar to our results. Interestingly, Mazumdar et al. described effective induction of cell death concomitant with PARP cleavage and Caspase 3 activity in human colon carcinoma cells upon GLI inhibition with GANT61, while upstream inhibition of SMO with cyclopamine was far less effective [48]. Cyclopamine also failed to induce significant apoptosis in human pancreatic carcinoma cells [41]. By contrast, colorectal cancer cells exhibited an increased apoptotic fraction when treated with 50 µM vismodegib [34], suggesting that a cell line-dependent Hh/GLI canonical and/or non-canonical signaling is responsible for induction of apoptotic cell death.

Data on an impact of Hh/GLI signaling on DNA damage response pathways, including base excision repair, nucleotide excision repair, non-homologous end joining (NHEJ), and homologous recombination (reviewed in Reference [27]), and own findings, of a modulation of DNA double-strand break repair by Survivin [26,28], prompted us to analyze DNA repair capacity in our cellular models. Vismodegib and radiation treatment enhanced residual radiation-induced DNA damage as assayed by γH2AX/53BP1 foci detection in both cell lines investigated (Figure 4) indicating that the cytotoxic effect is, at least in part, mediated by a hampered DNA damage response. These findings are concomitant with reports in colon adenocarcinoma showing an upregulation of γH2AX, activation of Ataxia telangiectasia mutated (ATM)/checkpoint kinase 2 (Chk2) upon GLI inhibition with GANT61 [48,49].

In conclusion, in the present study we provide preclinical evidence that inhibition of the Hh signaling pathway by vismodegib may increase therapeutic efficacy in both BCC and HNSCC if combined with ionizing radiation. Our findings thus support further preclinical studies on the complex underlying mechanisms and on extended clinical investigation of Hh pathway inhibition by radiation therapy to explore the tolerability, and clinical efficacy of this combination in these entities.

## 4. Materials and Methods

### 4.1. Cell Culture

A human basal cell carcinoma line BCC-1/KMC (BCC-1) was established by Chiang et al. [50] and kindly provided by the National Taiwan University (Taipei, Taiwan). SCC-25 (ATCC^®^ CRL1628™) human HNSCC were purchased from the American Type Culture Collection (LGC Standards, Wesel, Germany). The basaloid keratinocyte nature of these BCC-1 cells was verified by the presence of keratin 14 mRNA, as measured by performing real-time RT-PCR [51]. The BCC-1 cells were maintained in Gibco^TM^ RPMI medium 1640 (Thermo Fisher Scientific, Darmstadt, Germany) with 10% fetal bovine serum (FBS) superior (Merck, Darmstadt, Germany) and 100 units penicillin/mL/100 µg Streptomycin/mL (P/S) (Sigma-Aldrich, Munich, Germany) at 37 °C in a humidified atmosphere with 5% CO_2_. The SCC-25 cells were maintained in Dulbecco’s Modified Eagle’s Medium nutrient mixture F-12 Ham (Sigma-Aldrich), supplemented with 20% FBS and P/S at 37 °C in a humidified atmosphere with 5% CO_2_.

### 4.2. Vismodegib Treatment and Irradiation Procedure

Cells were treated with 5 to 40 µM vismodegib (Hölzel Diagnostika, Cologne, Germany) or with equivalent amounts of the solvent dimethyl sulfoxide (DMSO; AppliChem, Darmstadt, Germany) 24 h before irradiation or for indicated time periods. Irradiation with single doses of 2, 4, 6 or 8 Gy was performed using a linear accelerator (Elekta Synergy, Elekta, Crawley, UK) with 6 MV photon energy, 100 cm focus to isocentre distance and a dose rate of 6 Gy/min. To guarantee equal conditions control cells were kept in equivalent surroundings in the irradiation control room.

### 4.3. Cell Proliferation (MTS) Assay

BCC-1 cells (2500 cells per well) and SCC-25 cells (4000 cells per well) were plated in 96-well plates and cultured for 24 h before treatment with vismodegib. After 48 h of vismodegib treatment without and in combination with irradiation (4 Gy after 24 h), a colorimetric cell proliferation assay (CellTiter 96^®^ Aqueous One Solution Cell Proliferation (MTS) Assay, Promega, Mannheim, Germany) was carried out according to the manufacturer’s instructions. Absorbance at 490 nm (reference 570 nm) was recorded with a 96-well microplate reader (Infinite M200 Pro, TECAN, Männedorf, Switzerland). All experiments were carried out independently three to five times, each in quadruplicate.

### 4.4. Western Blot Analysis

According to the scheme shown in Figure 2A, cell lysis was accomplished with radio-immunoprecipitation assay (RIPA) buffer at 24 or 48 h after vismodegib treatment. Total cell lysates containing 40 µg of protein were prepared in sodium dodecyl sulfate (SDS) sample buffer, separated on 10% SDS polyacrylamide gels, and transferred to a nitrocellulose membrane (GE Healthcare, Munich, Germany) for immunodetection with the following specific primary antibodies: Anti-GLI1 (2534, Cell Signaling, Frankfurt, Germany), anti-Survivin (AF886, R&D Systems, Wiesbaden, Germany), anti p53 (DO-1; sc-126, Santa Cruz Biotechnology, Heidelberg, Germany), anti-PARP (9542) and anti-Caspase-3 (9662), both from Cell Signaling. For detection, blots were incubated with goat anti-rabbit (4050-05) or goat anti-mouse (1030-05) horseradish peroxidase-linked antibodies (Biozol, Eching, Germany) and finally visualized with chemiluminescence using Pierce ECL Western Blotting Substrate (Thermo Fisher Scientific) and the Odyssey Fc imaging system (LI-COR Biotechnology, Bad Homburg, Germany). To confirm equal loading of protein amounts, a β-actin antibody was used (A5441, Sigma-Aldrich). Densitometric analysis for GLI1 and Survivin protein content was performed with the Image Studio Lite version 5.0 software (LI-COR Biotechnology), normalized to β-actin expression and presented relative to DMSO-treated controls.

### 4.5. RNA Preparation and Quantitative Real-Time RT-PCR

Measurement of GLI1 or Survivin mRNA expression of BCC-1 and SCC-25 cells after 24 and 48 h treatment with vismodegib was accomplished by isolation of RNA using TRI Reagent^®^ (Sigma-Aldrich) according to the manufacturer’s recommendations. The time schedule for cell treatment and lysis is displayed in Figure 2A. Subsequently, RNA was reverse-transcribed with Omniscript^®^ Reverse Transcription Kit (Qiagen, Hilden, Germany) and quantitative real-time RT-PCR was performed using the StepOnePlus Real-Time PCR System (Thermo Fisher Scientific) and SYBR^®^ Green JumpStart^TM^ Taq ReadyMix^TM^ (Sigma-Aldrich). PCR assays were performed in triplicate according to the manufacturer’s instruction with the following PCR conditions/thermo-profile: 2 min at 94 °C, followed by 40 cycles of 15 s at 94 °C and 1 min at 54 °C (ribosomal protein L37A (RPL37A) and BIRC5) or 60 °C (GLI1). Relative gene expression was calculated using the 2^−∆∆*C*t^ method with the housekeeping gene RPL37A as reference and is displayed as mean values relative to DMSO-treated non-irradiated controls + standard deviation (SD). The sequences of the primer pairs were as follows: Survivin (gene name: BIRC5): BIRC5-fw 5′-CCCAGTGTTTCTTCTGCTTCAAG-3′, BIRC5-rev 5′-CAACCGGACGAATGCTTTTT-3′; (design: R. Wirtz, Stratifyer Molecular Pathology, Cologne, Germany; manufacturer: Eurofins Genomics, Ebersberg, Germany); GLI1: GLI1-fw: 5′-CTGTCCCATCCCGAACTCTC-3′, GLI1-rev: 5′-CCACCCATATCTCCCCTTCA-3′ (Integrated DNA Technologies (IDT), Leuven, Belgium); RPL37A: RPL37A-fw 5′-TGTGGTTCCTGCATGAAGACA-3′, RPL37A-rev 5′-GTGACAGCGGAAGTGGTATTGTAC-3′ [52] (manufacturer: Eurofins Genomics). For each data point two independent experiments were acquired and performed in duplicate.

### 4.6. Cell Cycle and Apoptosis Analysis

Analysis of cell cycle distribution and SubG1 population of BCC-1 and SCC-25 cells was performed with a CytoFlex flow cytometer (Beckman Coulter, Krefeld, Germany). Briefly, cells were plated in 6-well plates and treated with indicated amounts of vismodegib as described before. At 24 h after inhibitor treatment, the cells were irradiated (4 Gy, single dose; Figure 3A). A subsequent 24 h later, cells were trypsinized, washed with phosphate-buffered saline (PBS) (Thermo Fisher Scientific) and fixed with 80% ethanol at −20 °C for 10 min. After centrifugation (200× *g* for 5 min), cell pellets were resuspended in PBS containing 40 µg/mL propidium iodide (Sigma-Aldrich) and 40 µg/mL RNase A (Qiagen) and incubated for 30 min at 37 °C before measurement. Finally, cells were gated to exclude cell debris and analyzed by flow cytometry in linear mode by using the CytExpert Software (Beckman Coulter). Mean values and standard deviations were calculated by considering four independent experiments, each performed in duplicate.

### 4.7. Immunofluorescence Staining and Quantification of γH2AX/53BP1 Foci Formation

Analysis of residual DNA damage 24 h after irradiation was performed by quantification of γH2AX/53BP1-positive nuclear foci, a surrogate marker for DNA DSB, as described before [53]. Briefly, cells were plated on microscope cover slips and treated 24 h later with DMSO or with 10 or 40 µM vismodegib in 12-well plates (Greiner Bio-One, Frickenhausen, Germany) for 24 h. Cells were irradiated (0, 4 Gy) and 24 h thereafter fixed and permeabilized using 3.7% paraformaldehyde/0.25% Triton X-100 (AppliChem) diluted in PBS for 10 min. Blocking was performed in 5% bovine serum albumin (AppliChem) for 1 h and γH2AX/53BP1-positive foci were visualized with anti-γH2AX (clone JBW301, 05-636, Merck), anti-53BP1 (100-304, Novus Biologicals, Wiesbaden Nordenstadt, Germany) primary and fluorescent-dye conjugated secondary antibodies (Alexa Fluor 594 goat anti-mouse, Alexa Fluor 488 goat anti-rabbit, Thermo Fisher Scientific). Nuclei were stained with 4′,6-diamidino-2-phenylindole (DAPI) solution (AppliChem) and after mounting of cover slips with Vectashield mounting medium (Alexis, Grünberg, Germany), γH2AX/53BP1-positive nuclear foci were microscopically counted with an AxioImager Z1 microscope (Carl Zeiss, Jena, Germany). For each condition 50 nuclei were evaluated by considering three independent experiments performed in duplicate. Fluorescence images were acquired with an AxioImager Z1 microscope and AxioVision 4.6 software (Carl Zeiss).

### 4.8. 3D Colony Formation Assay

Evaluation of 3D clonogenic survival was performed as previously described [29]. Briefly, BCC-1 or SCC-25 cells were diluted in 0.5 mg/mL laminin-rich extracellular matrix (lrECM; BME Growth Factor Reduced PathClear) from Biozol at 500 or 400 cells per well. Vismodegib was applied 24 h thereafter at concentrations ranging from 5 to 40 µM. Controls were treated with equivalent amounts of DMSO solvent. 24 h after treatment, cells were irradiated (0–8 Gy, single doses) and 3D colonies >50 cells were counted microscopically 7 days after plating (Axio Vert.A1, 2.5-x objective, Carl Zeiss). Images of typical colony formation were acquired by phase contrast microscopy using an inverted microscope (Nikon TS100, Nikon, Düsseldorf, Germany). Plating efficiencies were measured as follows: Numbers of colonies formed after DMSO or vismodegib treatment /numbers of cells plated. Surviving fractions (SF) were calculated according to: Numbers of colonies formed / (numbers of cells plated (irradiated) × plating efficiency (non-irradiated, DMSO or vismodegib treated). For each point on the survival curves the mean surviving fraction from three independent experiments performed in triplicate repeats was calculated. Survival variables α, β were fitted according to the linear quadratic equation SF = exp [−α × *D* – *β* × *D*^2^] with *D* = dose (EXCEL software, Microsoft, version Office Professional Plus 2010, Redmond, WA, USA). A summary of radiation response variables and radiation-induced sensitizer enhancement ratios at 50% and 10% cell survival can be found in Table 1.

### 4.9. Statistical Analysis

Results are expressed as means ± SD calculated with GraphPad Prism (GraphPad Software, Inc., version 5, La Jolla, CA, USA) or EXCEL software. The unpaired two-tailed *t*-test was applied for statistical analysis (EXCEL software), while a *p*-value < 0.05 was considered statistically significant.

## Figures and Tables

**Table 1:** Radiation response variables of vismodegib-treated BCC-1 and SCC-25 cells.

Cell Line Treatment	Plating Efficiency [%]	α [Gy^−1^]	β [Gy^−2^]	Radiation Dose at 50% Cell Survival [Gy]	Sensitizer Enhancement Ratio (versus DMSO Control)	Radiation Dose at 10% Cell Survival [Gy]	Sensitizer Enhancement Ratio (versus DMSO Control)
BCC-1							
DMSO	23.29 ± 3.39	0.0428	0.0307	4.10		7.99	
5 µM Vism.	25.00 ± 3.67	0.2348	0.0074	2.72	1.51	7.85	1.02
10 µM Vism.	24.29 ± 4.84	0.2044	0.0147	2.82	1.45	7.37	1.08
40 µM Vism.	21.11 ± 1.54	0.2724	0.0084	2.37	1.73	6.96	1.15
SCC-25							
DMSO	28.56 ± 2.52	0.0849	0.0290	3.64		7.57	
5 µM Vism.	29.44 ± 1.67	0.2479	0.0138	2.46	1.48	6.75	1.12
10 µM Vism.	31.53 ± 5.59	0.3189	0.0104	2.04	1.79	6.03	1.25
40 µM Vism.	22.39 ± 2.43	0.3152	0.0143	2.02	1.81	5.79	1.31

The linear quadratic equation (SF = exp [*−α* × *D − β* × *D*^2^]) with fitted α and β values of the individual survival curves was transformed to calculate radiation-induced cytotoxicity enhancement factors at 50% and 10% cell survival versus DMSO controls. D, radiation dose [Gy]; DMSO, control cells treated with equivalent amounts of DMSO; SF, surviving fraction; Vism., vismodegib-treated cells using indicated concentrations.

## Supplementary Materials

Supplementary materials can be found at http://www.mdpi.com/1422-0067/19/9/2485/s1.

## Abbreviations

3D	Three-dimensional
53BP1	p53-binding protein 1
BCC	Basal cell carcinoma
DSB	Double-strand break
GLI	Glioma-associated oncogene homologue 1
Hh	Hedgehog
HNSCC	Head and neck squamous cell carcinoma
IAP	Inhibitor of apoptosis protein
PARP	Poly ((adenosine diphosphate)ADP-ribose) polymerase
PTCH1	Patched homologue 1
SCC	Squamous cell carcinoma
SMO	Smoothened
SUFU	Suppressor of fused
