# Supplementary material for: Radiation Sensitization of Basal Cell and Head and Neck Squamous Cell Carcinoma by the Hedgehog Pathway Inhibitor Vismodegib
Source: Int J Mol Sci. 2018 Aug 23;19(9):2485. doi: 10.3390/ijms19092485 (PMC6164565; doi:10.3390/ijms19092485)
Supplement: Supplementary file 1 [file ijms-19-02485-s001.zip › ijms-348884-supplementary.docx]

**Table S1.** Vismodegib significantly radiosensitizes 3D-cultured BCC-1 and SCC-25 cells to ionizing radiation.

|  | ***p-*value versus DMSO-treated cells** | | | |
| --- | --- | --- | --- | --- |
| **Cell line  treatment** | **Radiation dose 2 Gy** | **Radiation dose 4 Gy** | **Radiation dose 6 Gy** | **Radiation dose 8 Gy** |
| BCC-1 |  |  |  |  |
| 5 µM Vism. | 0.0029** | 0.0001** | 0.0056** | 0.0927 |
| 10 µM Vism. | 0.0000** | 0.0019** | 0.0069** | 0.0163* |
| 40 µM Vism. | 0.0024** | 0.0002** | 0.0037** | 0.0089** |
| SCC-25 |  |  |  |  |
| 5 µM Vism. | 0.2755 | 0.0112* | 0.0147* | 0.1168 |
| 10 µM Vism. | 0.1234 | 0.0068** | 0.0022** | 0.0084** |
| 40 µM Vism. | 0.0399* | 0.0060** | 0.0021** | 0.0047** |

The unpaired two-tailed *t*-test was applied for statistical analysis (EXCEL software) of vismodegib- versus DMSO-treated cells (*n* = 3) at indicated radiation doses and a **p*-value < 0.05 was considered statistically significant, while a ***p*-value < 0.01 was considered as highly statistically significant. BCC, basal cell carcinoma; SCC, squamous cell carcinoma; Vism., vismodegib-treated cells using indicated inhibitor concentrations.
