# Supplementary figures and images for: Radiation Sensitization of Basal Cell and Head and Neck Squamous Cell Carcinoma by the Hedgehog Pathway Inhibitor Vismodegib
Source: Int J Mol Sci. 2018 Aug 23;19(9):2485. doi: 10.3390/ijms19092485 (PMC6164565; doi:10.3390/ijms19092485)

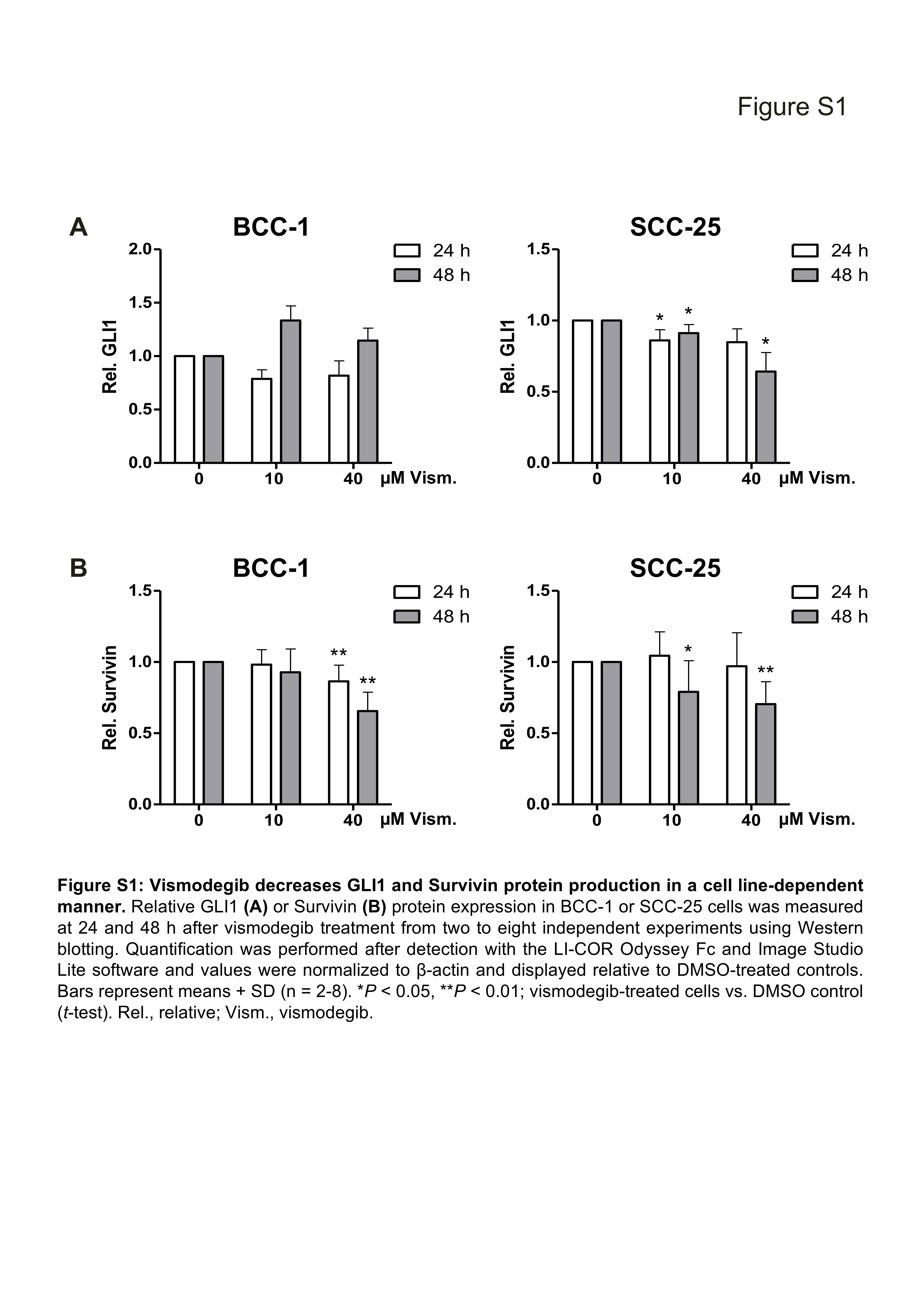

Supplement: Supplementary file 1 [file ijms-19-02485-s001.zip › Figure_S1_rev.jpg]

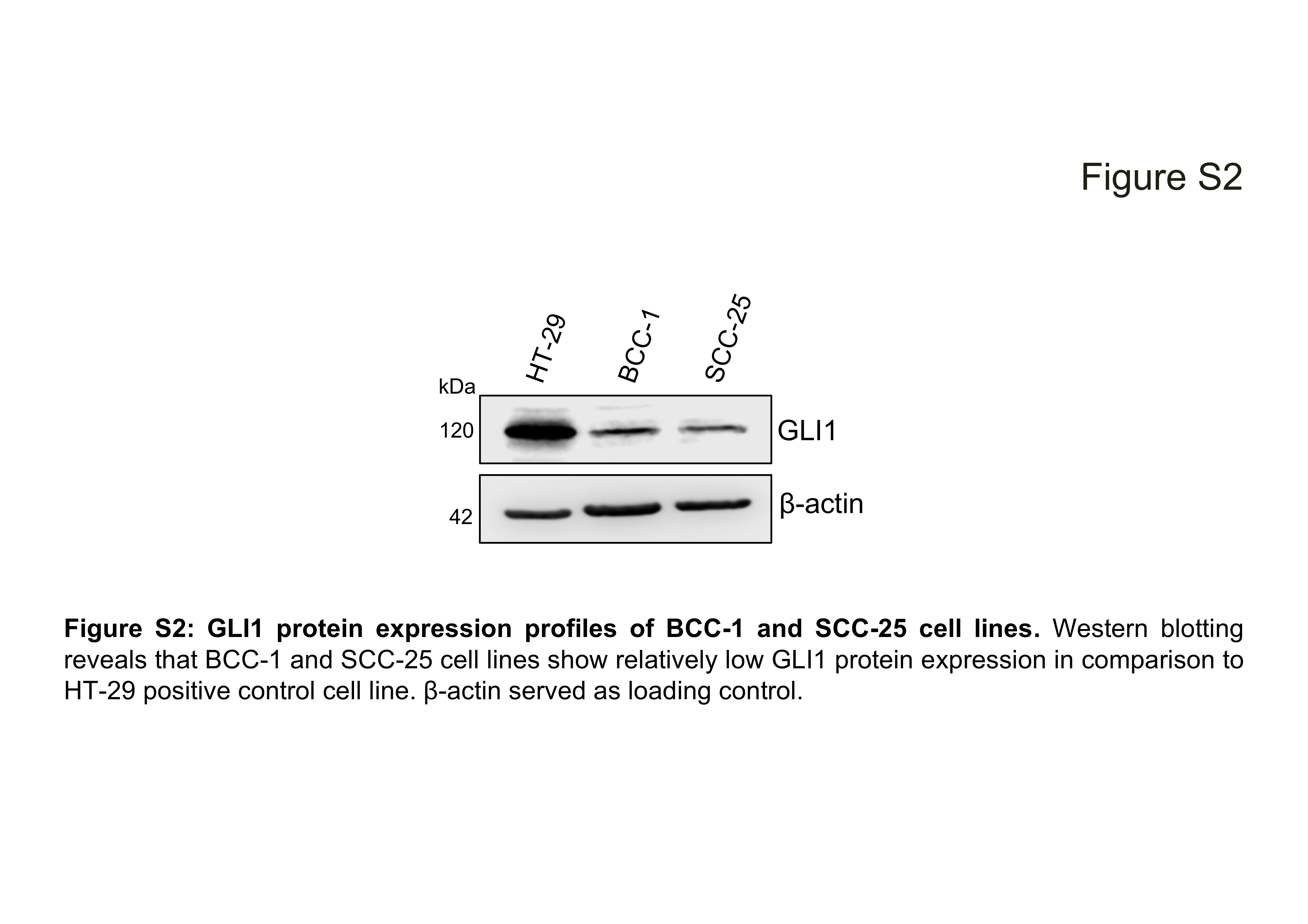

Supplement: Supplementary file 1 [file ijms-19-02485-s001.zip › Figure_S2_rev.jpg]

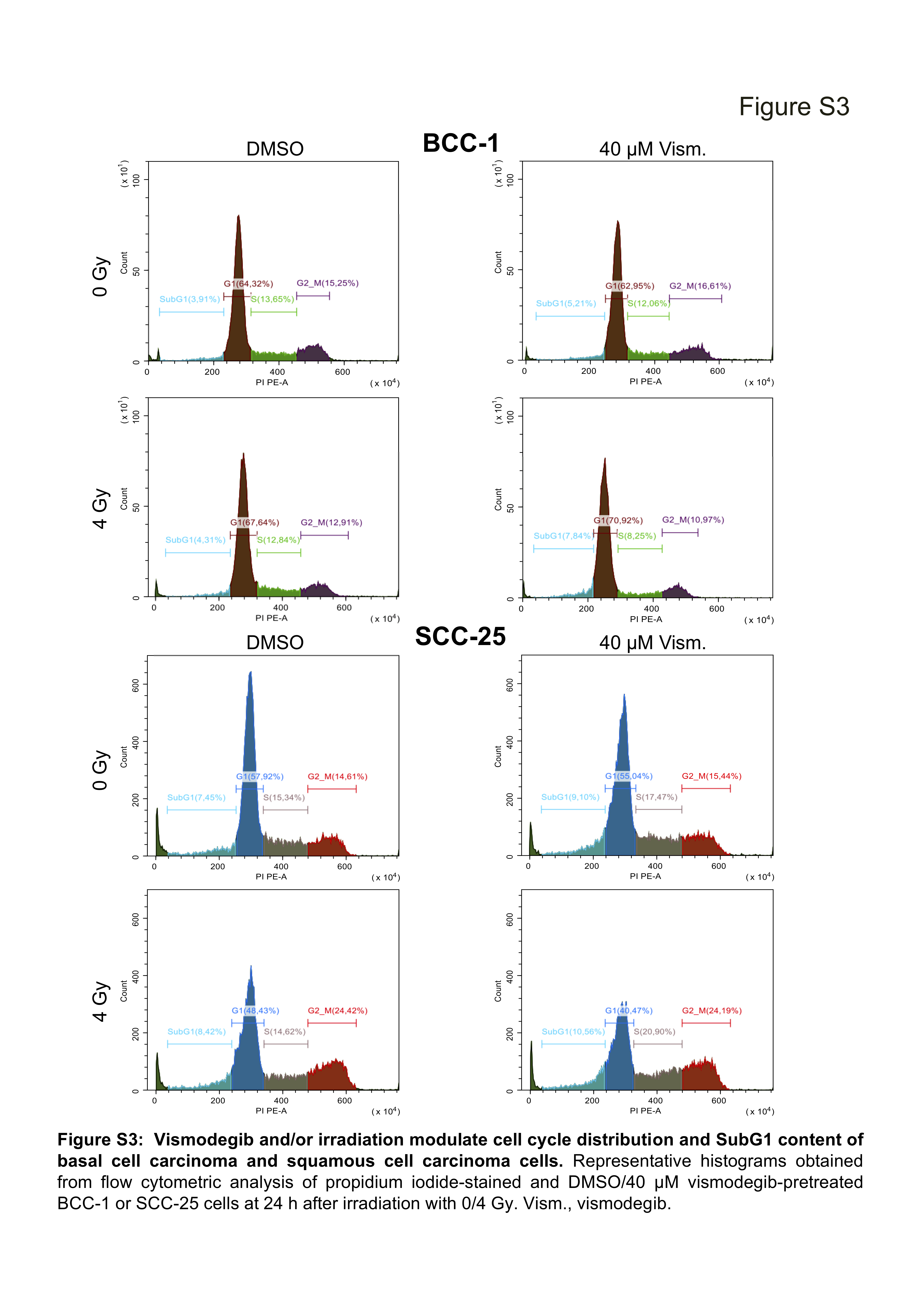

Supplement: Supplementary file 1 [file ijms-19-02485-s001.zip › Figure_S3_rev.jpg]
